# Coinfection of diarrheagenic bacterial and viral pathogens in piglets of Northeast region of India

**DOI:** 10.14202/vetworld.2019.224-230

**Published:** 2019-02-09

**Authors:** Hosterson Kylla, Tapan K. Dutta, Parimal Roychoudhury, Prasant K. Subudhi

**Affiliations:** 1 Department of A.H and Veterinary, Disease Investigation Office, Meghalaya, Shillong, India; 2 Department of Veterinary Microbiology, Central Agricultural University, Aizawl, Mizoram, India

**Keywords:** coinfection, *Escherichia coli*, *Picobirnavirus*, piglets, *Rotavirus*, *Salmonella*

## Abstract

**Aim:**

This study aimed to study the prevalence of the coinfection of enteric bacterial and viral pathogens, namely *Escherichia coli, Salmonella*, *Rotavirus*, and *Picobirnavirus* from fecal samples of pre-weaned piglets in Northeast region of India.

**Materials and Methods:**

A total of 457 fresh fecal samples were collected from piglets under 9 weeks old during 2013-2015 from organized (n=225) and unorganized (n=232) farms of Manipur, Meghalaya, Mizoram, and Nagaland. Samples were collected from diarrheic (n =339) and non-diarrheic (n=118) piglets including local indigenous (n=130) and crossbreed (n=327) piglets in different seasons during the study period. The samples were processed for the isolation of *E. coli* and *Salmonella* and detection of their putative virulence genes by polymerase chain reaction (PCR). Samples were also processed for the detection of *Rotavirus* and *Picobirnavirus* by RNA-polyacrylamide agarose gel electrophoresis and reverse transcriptase-PCR (RT-PCR).

**Results:**

A total of 11 (2.40%) samples were found positive for two or more coinfecting enteric bacterial and viral pathogens. All the 11 positive fecal samples were recovered from diarrheic piglets. *Salmonella* Typhimurium (enterotoxin, *stn* gene) and *Picobirnavirus* genogroup 1 were found to be more frequent as coinfecting agents. Coinfection was recorded higher in unorganized (3.87%) compared to organized farm (0.88%). Again, higher detection was recorded in crossbreed (2.75%) than local indigenous piglets (1.53%). The occurrence of coinfection was found to be more common during summer (4.68%) followed by winter (2.27%) season.

**Conclusion:**

The present study highlighted the significance of *E. coli*, *Salmonella*, *Rotavirus*, and *Picobirnavirus* as important diarrheagenic pathogens causing coinfection in piglets in Northeast region of India. Probably, this is the first systematic study of the coinfection of four important diarrheagenic bacterial and viral agents associated with piglet diarrhea in India.

## Introduction

Piggery is a promising venture with vast potential in Northeast region, and the region has the highest pig population in the country with 3.95 million compared to 10.29 million pig population in the whole country [1]. Many swine diseases and ailments affect the productivity particularly pre-weaned piglets, and it was estimated that diarrhea alone accounts for a reduction of 8-14 g/day in average daily weight gain in the 1^st^ week of life [2]. Every year, huge losses in terms of health and productivity are attributed to piglet’s diarrhea worldwide. The mechanism of viral and bacterial coinfection contribution to disease augmentation was first explained while studying role of secondary bacterial infections during influenza pandemics [3] in which many bacterial agents were associated, but *Staphylococcus aureus*, *Streptococcus pneumoniae*, and *Haemophilus influenzae* are the major bacteria associated in concurrent infection with influenza virus [4]. Researchers have demonstrated that a single susceptible host is attacked and often infected by two or more pathogen species or strains [5,6], in which case coinfecting pathogens may act synergistically, resulting in even greater pathogenesis and such involvement may contribute to the overall disease burden in host species both individually and together in a herd [7]. Such concurrent infections not only augment the clinical course of infections but can also influence host immune responses to pathogens [8,9] and eventually affect the effectiveness of many disease control program in a state or country [10]. Coinfecting pathogens in any species may cause severe diarrhea than infection with a single pathogen alone [11]. Most often concurrent infection with multiple pathogens is common particularly among young children with diarrhea and often followed a long severe course and manifestation than infection with a single causal pathogen [12,13].

Infectious diarrhea of neonatal animals is one of the most common and economically devastating conditions encountered in the livestock industry, some of the diarrheas are self-limiting, some are associated with high morbidity, and others are associated with high mortality [14]. Diarrhea remains the second leading cause of death in young children worldwide, accounting for 1.3 million deaths annually and it alone amounts to an estimated 4.1% of the total global burden of disease [15,16]. In food animal species, an array of infectious agents including bacteria, viruses, and parasites cause acute and chronic diarrhea in piglets including *Campylobacter* spp., *Clostridium* *perfringens*, diarrheagenic *Escherichia coli*, *Salmonella, Rotavirus*, *Coronaviruses* (transmissible gastroenteritis virus), porcine epidemic diarrhea virus, as well as by nematode and protozoan parasites [17,18]. Such enteric infections lead to gastroenteritis causing life-threatening implication, especially to newborns. Although there are many diarrheagenic pathogens, most studies have been focused only on one pathogen or on few causal agents, as a result, a clear concept of understanding of the relative importance of such causal pathogens and other related factors may have strong biases.

Porcine bacterial and viral enteric pathogens play an important role in terms of zoonoses, drugs resistance, morbidity, and mortality of pigs of different age groups, particularly young pigs, and overall in the hindrance of piggery development program. Only limited work has been carried out in India regarding coinfection of bacterial and viral enteric pathogens. Keeping in view the importance of such pathogens in piglets, the aim of the present research study was to assess the prevalence and molecular characterization of *E. coli*, *Salmonella*, *Rotavirus*, and *Picobirnavirus* as coinfecting pathogens from piglets of organized and unorganized farms of Manipur, Meghalaya, Mizoram, and Nagaland states of India.

## Materials and Methods

### Ethical approval

The present research work was meant for prevalence study of enteric organisms and no animals were harm during the study. Samples were collected as per standard sample collection procedure.

### Collection of fecal samples, animals, and sampling

**Figure-1:**
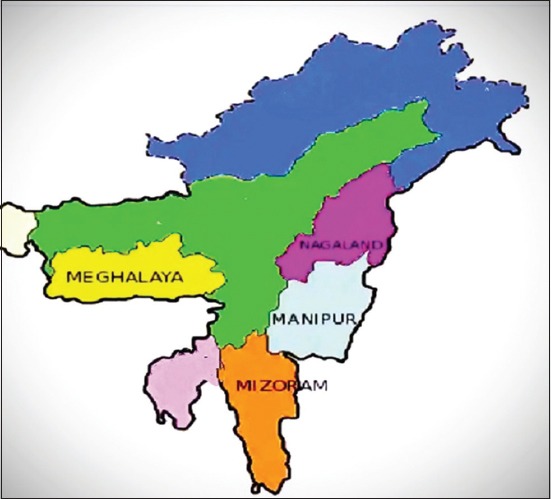
A map representing the four Northeast states of India in which representative sampling were collected for the study. (Source: http:/www.mea.gov.in/india-at-glance.htm)

A total of 457 fresh fecal samples were collected from piglets under 9 weeks old from organized (n=225) and unorganized (n=232) farms of four Northeastern Hilly states of India (Figure-1), namely Manipur (n=108), Meghalaya (n=124), Mizoram (n=120), and Nagaland (n=105). Samples were collected from diarrheic (n=339) and non-diarrheic (n=118) piglets including indigenous local (n=130) and crossbreed (n=327) piglets. Samples were collected in four different seasons of the year, namely spring (n=93), summer (n=128), autumn (n=104), and winter (n=132) during June 2013-May 2015.

Fecal samples were collected and transported to laboratory under cold chain. All the samples were processed for the isolation and identification of *E. coli* and *Salmonella* and the detection of enteric viruses including *Rotavirus* and *Picobirnavirus* by RNA-polyacrylamide agarose gel electrophoresis (PAGE) and reverse transcriptase-polymerase chain reaction (RT-PCR).

### Bacteriological screening of clinical specimens

*E. coli* and *Salmonella* were cultured aerobically. For *E. coli* culture, samples were streaked directly on MacConkey agar (HiMedia, India) and characteristic pink-colored colonies were streaked on eosin methylene blue agar plates followed by overnight incubation at 37°C. Colonies with characteristic metallic sheen were studied for their characteristics. For *Salmonella* culture, about 2 g of fecal sample was inoculated in 5 ml Rappaport Vassiliadis broth (HiMedia, India) for enrichment and incubated at 37°C for 18-20 h. Culture was then streaked on Xylose lysine deoxycholate agar (HiMedia, India) and incubated at 37°C overnight. Typical black centered with bright-edged colonies were selected and streaked on brilliant green agar (BGA) plates. Colonies with pink color on BGA agar were then studied for their characteristics. All the characteristic *E. coli* and *Salmonella* isolates were subjected to identification by standard biochemical tests such as indole test, methyl red test, Voges-Proskauer test, citrate utilization test, and hydrogen sulfide production on triple sugar iron agar [19]. All the pure isolates were stored in glycerol at −70°C for further use.

### Detection of *Rotavirus* and *Picobirnavirus*

The fecal samples were screened for the presence of *Rotavirus* and *Picobirnavirus* by RNA-PAGE analysis following standard protocol. In brief, samples were diluted in phosphate-buffered saline (pH 7.4) to prepare a 10% (w/v) fecal suspension. Clarified supernatant was collected and processed for RNA extraction using Trizol method [20]. The extracted RNA was subjected to RNA-PAGE followed by silver staining as per standard procedure [21]. *Rotavirus* and *Picobirnavirus* were also detected by RT-PCR. Detection of *Rotavirus* Groups A and C was performed by targeting VP7 gene [22] and VP6 gene [23], respectively. For the detection of *Picobirnavirus* genogroup I (GGI) [24] and GGII [25], specific primers were used. Details of oligonucleotides and PCR conditions used in the present study are given in Table-1 [22-29].

**Table-1:** Oligonucleotide primers used in the present study.

Primer	Sequences (5’- 3’)	Annealing temperature	Amplicon size (bp)	References
Rota A (VP7)	(F) TTGACTAARGGRTGGCCAACWGG	55°C	540	[22]
	(R) TCGCATCATHCKYTCNGTTTGTGG			
Rota C (VP6)	(F) CTCGATGCTACTACAGAATCAG	46°C	356	[23]
	(R) AGCCACATAGTTCACATTTCATCC			
PBV (GG1)	(F) TGGTGTGGATGTTTC	48°C	201	[24]
	(R) A(G,A)TG(C,T)TGGTCGAACTT			
PBV (GGII)	(F) GACCGGTWTGGATGTTTCCGATG	56°C	369	[25]
	(R) GTATCTGTGCTGGSCGCAT			
*eaeA*	(F) GACCCGGCACAAGCATAAGC	61°C	384	[26]
	(R) CCACCTGCAGCAACAAGAGG			
*st×*1	(F) ATAAATCGCCATTCGTTGACTAC	61°C	180	[26]
	(R) AGAACGCCCACTGAGATCATC			
*st×*2	(F) GGCACTGTCTGAAACTGCTCC	61°C	253	[26]
	(R) TCGCCAGTTAATCTGACATTCTG			
*hlyA*	(F) GCATCATCAAGCGTACGTTCC	61°C	534	[26]
	(R) AATGAGCCAAGCTGGTTAGCT			
*stn*	(F) TTGTGTCGCTATCACTGGCAACC	61°C	617	[27]
	(R) ATTCGTAACCCGCTCTCGTCC			
*invA*	(F) ACCACGCTCTTTCGTCTGG	60°C	941	[28]
	(R) GAACTGACTACGTAGACGCTC			
*pef*	(F) TGTTTCCGGGCTTGTGCT	57°C	700	[29]
	(R) CAGGGCATTTGCTGATTCTTCC			

GG1=Genogroup 1, GGII=Genogroup II

### Detection of bacterial virulence genes by PCR

DNA lysate for PCR analysis was prepared by standard boiling and snap chilling method. Detection of the putative virulence genes of enteropathogenic *E. coli* (EPEC, *eaeA*), Shiga-toxigenic *E. coli* (STEC, *stx_1_*, *stx_2_*), and enterohemorrhagic *E. coli* (*hlyA*) was evaluated by multiplex PCR [26].

In case of *Salmonella* isolates, the detection of enterotoxin (*stn*) [27], invasive (*invA*) [28], and plasmid efficacy fimbrial (*pef*) [29] genes was carried out by specific PCR assay in a thermal cycler (Eppendorf, Germany). Oligonucleotides and PCR conditions used in the study are depicted in Table-1 [22-29].

### Agarose gel electrophoresis

All amplified products were separated by 1% agarose gel in 1X Tris-borate-EDTA buffer by electrophoresis and stained with ethidium bromide (0.5 µg/ml). Standard molecular size marker (100 bp DNA ladder) was included in each gel. DNA fragments were observed by ultraviolet transilluminator and photographed in a gel documentation system (Alpha Imager, Germany).

### Serotyping of isolates

*Salmonella* isolates were serotyped at National *Salmonella* and *Escherichia* Centre, Kasauli, Himachal Pradesh, India, and the National *Salmonella* Centre, Indian Veterinary Research Institute, Bareilly, Uttar Pradesh, India.

## Results

Bacterial and viral diarrheagenic pathogens are persistent in livestock population in India. In the present study, samples positive for coinfection with *E. coli, Salmonella*, *Rotavirus* A, and *Picobirnavirus* GG1 were detected from diarrheic fecal samples (n=339) only; whereas, no coinfection of organisms was detected from 118 non-diarrheic fecal samples collected during 2013-15.

A total of 11 (2.40%) fecal samples from Northeast states were found positive for more than one pathogen as depicted in Table-2. Of the overall 11 positive samples, two samples from Manipur were found positive for coinfection of EPEC and *Picobirnavirus* GG1. In case of coinfection from samples of Meghalaya, a total of five diarrheic samples were found positive for at least two enteric pathogens. One fecal sample was found positive for EPEC and *Rotavirus* A; two samples were positive for both *Salmonella* Typhimurium (*stn*) and *Picobirnavirus* GGI; one sample was found positive for EPEC and *Picobirnavirus* GG1, whereas, in one diarrheic sample, three enteric pathogens, namely *Picobirnavirus* GG1, EPEC, and *Salmonella* rough strain (*stn*) were detected harboring associated virulence genes.

**Table-2:** Coinfection of enteric bacterial and viral pathogens in diarrheic fecal samples.

State	Samples positive for >1 gene	Coinfecting pathogens with associated genes
Manipur	n=2	*E. coli* (*eaeA*)+*Picobirnavirus* GG1
Meghalaya	n=1	*E. coli (eaeA*)+*Rotavirus* A
	n=1	*E. coli* (*eaeA*)+*S. rough* (*stn*)+*Picobirnavirus* GG1
	n=2	*S.* Typhimurium (*stn*)+*Picobirnavirus* GG1
	n=1	*E. coli* (*eaeA*)+*Picobirnavirus* GG1
Mizoram	n=1	*S.* Typhimurium (*invA*)+*E. coli* (*st×_2_*)+*Picobirnavirus* GG1
	n=2	*S.* Typhimurium (*stn*)+*Picobirnavirus* GG1
Nagaland	n=1	*S.* Hiduddify (*stn*)+*E. coli* (*st×_2_*)
Total	n=11	

GG1=Genogroup 1, *S.* Typhimurium=*Salmonella* Typhimurium, *E. coli=Escherichia coli*

In Mizoram, a total of three samples were positive for coinfection with at least two pathogens. One diarrheic sample was found positive for STEC (*stx_2_* gene), *S*. Typhimurium (invasive, *invA* gene), and *Picobirnavirus* GG1; and two diarrheic fecal samples were found positive for both *S*. Typhimurium (*stn*) and *Picobirnavirus* GG1. However, in diarrheic samples from piglets of Nagaland, only one sample was found positive for *S*. Typhimurium (*stn*) and STEC (*stx_2_*).

Among the pathogens under study, coinfection with either *Picobirnavirus* GG1 was found to be more frequent (n=8). In all the positive samples with *Picobirnavirus* GG1 were found as coinfective with enteric bacterial pathogens either EPEC, STEC, or *Salmonella*. However, *Rotavirus* type A (RVA) was detected only in one diarrheic sample as coinfection with EPEC. Five diarrheic samples were found to be coinfective of EPEC with other infectious agents, namely *Salmonella*, RVA, and *Picobirnavirus* GG1. Shiga-toxigenic *E. coli* STEC (*stx_2_*gene) were detected as coinfective strain with *Salmonella* and *Picobirnavirus* GG1 in two diarrheic samples under the study. Again, seven diarrheic samples from North Eastern states were found to be positive for *Salmonella* spp. as one of the coinfective agents with other enteric pathogens, in which the most frequent *Salmonella* serotype involved was Typhimurium (Table-2).

In total, 9 (3.87%) samples harboring pathogens involved as coinfection in piglets was detected from unorganized farms, whereas only 2 (0.88%) samples were from organized farms. Again, of the 11 positive samples, crossbreed piglets recorded higher detection of mixed infection (9; 2.75%) than local indigenous piglets (2; 1.53%). The samples in the present study were collected during different seasons of the year. The occurrence of coinfection was found to be most common during summer season (n=6; 4.68%), followed by winter (n=3; 2.27%), spring (n=1; 1.07%), and autumn (n=1; 0.96%), respectively.

## Discussion

In our study, coinfection of at least two infectious pathogens is recorded in 2.4%, whereas higher prevalence was recorded by other researchers. In a study by Mesonero-Escuredo *et al*. [30] recorded that 43.1% of samples were positive for more than one pathogen either bacterial or viral pathogens in pig neonatal diarrhea cases in Spain with *C. perfringens* type A being involve in all cases. However, Katsuda *et al*. [31] noted that infectious diarrhea in newborn piglets is usually related to the presence of a single pathogen and mixed infections are considered less common. In another study by Zhang *et al*. [32] recorded 4.3% of positive cooccurrences of enteric pathogens in sick children in Southwest China where *E. coli* and *Norovirus* were the predominant coinfective pathogens. These reports suggested that coinfection of pathogens varies from one region to another geographical region. Neonatal diarrhea, particularly in young piglets, is often complex with a mixture of infectious agents and other factors such as passive immunity transferred by colostrum and milk, environmental temperature and humidity [33,34], and managemental factor [35], all contributing to its manifestation.

In the present study, *Picobirnavirus* GG1 was recorded as highest cases as coinfecting agent. *Picobirnaviruses* are generally regarded as diarrheagenic viruses because most of their detection is associated with virus shed in feces and have been detected in animals and human patients with and without gastroenteritis and mostly coinfected with other enteric viruses such as *Rotavirus, Astrovirus, Caliciviru*s, and *Coronaviru*s [36,37].

Many researchers [38-41] reported that RVA is the most common cause of viral diarrhea in neonatal pigs compared to Type C, and other workers have detected porcine *Rotavirus* in diarrheic fecal samples in nursing, weaning, and post-weaning pigs either alone or in combination of other enteric pathogens such as *E. coli*, *Salmonella*, and *Adenovirus* [42,43]. *Rotavirus* A is only often detected as the sole infectious agent than as coinfection in cases of neonatal diarrhea [31,44]. This is in agreement with our finding in which RVA was detected only in one diarrheic sample as coinfection with EPEC. Although it is clear that RVA has an overwhelming impact on diarrhea illnesses in children, coinfection with other enteric pathogens appears to aggravate diarrhea severity. These findings should serve as evidence for public health services when planning and developing intervention programs [32].

Diarrheagenic *E. coli* are important common bacterial pathogen involve in piglets diarrhea. According to Fairbrother and Gyles [33], the main pathotype of *E. coli* responsible for intestinal disease in pigs is enterotoxigenic *E. coli* and EPEC also known as attaching and effacing *E. coli*. Coinfection of EPEC with other infectious agents indicates that EPEC potentially accelerates gastroenteritis and is one of the main pathogens involved in coinfection with other diarrheagenic bacterial or viral pathogens. Shiga-toxigenic *E. coli* was detected as coinfective strain in two diarrheic samples. The strain is one of the leading causes of diarrhea in the developing world and importantly in Northeast India, and they are commonly recovered from diarrheic feces of food-producing animals including porcine [45,46].

Salmonellosis is one of the economically important enteric and septicemic diseases associated with morbidity and even mortality in farm animals [47]. In our study, *S*. Typhimurium was the most frequent serovar involved in coinfection; however, this serovar is not host-specific serovar in pigs; hence, the finding is an indication of a public health and zoonotic concern. The close monitoring of such concurrent infection in porcine is important not only in terms of production but also from a public health point of view.

In the present study, higher detection of coinfection was recorded from unorganized farms, compared to organized farms. The reason for variation probably might be due to different management being practice from farm to farm, particularly in case of *Picobirnaviruses* which can enter and transmit mainly through sewage and untreated water in the farm [48]. Crossbreed piglets recorded higher detection of mixed infection than local indigenous piglets. Although the variation is not very significant, it may be possible that the local non-descriptive piglets possess better immunity against natural infection than crossbreed animals. In addition, the weaning age of piglets of the local animals is generally 8-10 weeks in comparison with the crossbreed animals in organized farms (within 6 weeks). Maternal immunity might also play an important role in resisting the infection in piglets, particularly in indigenous pigs. In the same region in a study of diarrheagenic pathogens, reported a higher prevalence rate of *Picobirnavirus* and STEC in crossbreed than indigenous piglets population [24,46]. In our study, samples collected during different seasons showed that coinfection of enteric pathogens was found to be most common during the summer (June-August). However, due to paucity of reports regarding seasonal variation of coinfection, hence, this did not allow us to compare our results. The humid climatic condition during summer with persistent rainfall in Northeast region may be an important reason for entry and transmission of infection among the pig population. Such climatic condition also allows suitable environment for persistent enteric pathogens such as *E. coli*, *Salmonella*, *Rotavirus*, and *Picobirnavirus* to cause diarrhea in piglets.

Diarrhea or scour has been recognized as one of the most common ailments causing deaths of young animals including piglets, but it is very often a neglected problem in most farming system. In the present study, a total of 11 diarrheic fecal samples were detected for a combination of at least two different enteric pathogens which are suggestive of the fact that a number of enteric pathogens may cause diarrhea either singly or in combination. Considering the importance of *E. coli*, *Salmonella*, *Rotavirus*, and *Picobirnavirus* as important diarrheal causing agents in animal species and human, hence, there is a possibility of transmission between porcine and human population, particularly in rural tribal areas in North East Hilly region of India were people reared two or three pigs in close proximity with human habitation and most share common drinking water.

## Conclusion

Only limited work has been carried out in India about coinfection of diarrheagenic bacterial and viral agents in porcine. Hence, the present study provides updated information and highlighted the significance of *E. coli*, *Salmonella*, *Rotavirus*, and *Picobirnavirus* as important diarrheagenic pathogens causing coinfection in piglets, where such data are scarce. In conclusion, it may be stated that coinfections with either of the four enteric bacterial and viral pathogens persist and associated with the piglet diarrhea in Northeast region of India, particularly with *Picobirnavirus* and *S*. Typhimurium. Higher prevalence is recorded from unorganized compared to organized farm and higher detection in crossbreed compared to the local indigenous pigs with higher detection during the summer season. This probably the first systematic study of the prevalence of the coinfection of important diarrheagenic bacterial and viral pathogens associated with piglet diarrhea in India.

## Authors’ Contributions

TKD and HK designed and planned the research work. HK collected the samples and executed the isolation, biochemical, and molecular characterization work and carried out the antibiotic sensitivity assay of all isolates. HK, TK, PR, and PKS analyzed the data and monitored the results and assay. All authors contributed equally in the preparation and revision of the manuscript. All authors read and approved the final manuscript.
